# Integrated micro-optofluidic platform for real-time detection of airborne microorganisms

**DOI:** 10.1038/srep15983

**Published:** 2015-11-02

**Authors:** Jeongan Choi, Miran Kang, Jae Hee Jung

**Affiliations:** 1 Center for Environment, Health, and Welfare Research, Korea Institute of Science and Technology, Seoul 136-791, Republic of Korea

## Abstract

We demonstrate an integrated micro-optofluidic platform for real-time, continuous detection and quantification of airborne microorganisms. Measurements of the fluorescence and light scattering from single particles in a microfluidic channel are used to determine the total particle number concentration and the microorganism number concentration in real-time. The system performance is examined by evaluating standard particle measurements with various sample flow rates and the ratios of fluorescent to non-fluorescent particles. To apply this method to real-time detection of airborne microorganisms, airborne *Escherichia coli*, *Bacillus subtilis*, and *Staphylococcus epidermidis* cells were introduced into the micro-optofluidic platform via bioaerosol generation, and a liquid-type particle collection setup was used. We demonstrate successful discrimination of SYTO82-dyed fluorescent bacterial cells from other residue particles in a continuous and real-time manner. In comparison with traditional microscopy cell counting and colony culture methods, this micro-optofluidic platform is not only more accurate in terms of the detection efficiency for airborne microorganisms but it also provides additional information on the total particle number concentration.

Airborne microorganisms (or bioaerosols), including viruses, bacteria, and fungal spores, are associated with a wide range of health effects and environmental issues^1^. Because of the low settling velocities due to the small size of particles (~20 nm to 100 μm), such bioaerosols may be suspended in the atmosphere for a prolonged period, and can be carried over large distances^2^. Therefore, there is significant potential for inhalation into the human respiratory system, which has health consequences^3^. There have been many studies on the health effects of exposure to harmful bioaerosols, such as infectious diseases, pneumonia, asthma, and allergies^4^,^5^. The increased use of buildings that are isolated from external ventilation brings about increased risk of exposure to bioaerosols^6^,^7^. Outdoor workers in the recycling industry are often exposed to high concentrations of bioaerosols^8^,^9^. The recent pandemic outbreaks of H1N1 influenza A and severe acute respiratory syndrome (SARS) demonstrate the importance of bioaerosol monitoring and control^10^,^11^,^12^.

Growing concerns over airborne microorganisms have led to demand for accurate and reliable monitoring systems to protect humans from exposure to harmful microorganisms. Several epidemiological studies have demonstrated links between airborne microorganisms and health problems. The airborne concentration of culturable microorganisms (i.e., colony-forming units (CFUs) per cubic meter of air) is a particularly widely used indicator, and is a simple parameter that can be used in the quantification of air quality^4^,^7^,^13^,^14^. For example, the World Health Organization (WHO) has suggested guidelines regarding the maximum allowable airborne fungal count (i.e., <500 CFU/m^3^)^15^.

Microscopy and culture techniques are conventionally used to analyze bioaerosols. Microscopy methods can determine the total bioaerosol concentrations based on the use of hemocytometers; however, this is time-consuming and operator-dependent. Culturing methods require a growth medium, which may not satisfy the specific growth requirements of all species of interest, and also requires prolonged incubation (i.e., >24 hours)^16^. Recently, detection and enumeration of airborne microorganisms has been accomplished using techniques based on polymerase chain reaction (PCR) and enzyme-linked immunosorbent assays (ELISA), which are highly sensitive and quantitative techniques^17^,^18^,^19^,^20^,^21^. However, additional pretreatment processes (such as particle condensation/purification), and elaborate sample handling by well-trained operators in a clean environment are required^22^. Although these PCR and ELISA techniques have been applied to microfluidic chips to simplify the treatment processes,^23^,^24^,^25^ most such microfluidic systems have been developed as disposable chips for point-of-care diagnosis of target microorganisms, which often leads to a limited number of samples or poor accuracy or rates of detection^26^.

Novel real-time methods for the rapid detection and continuous monitoring of airborne microorganisms have the potential to address the public health problems associated with bioaerosols. One of the most frequently used real-time detection techniques for detection of airborne microorganisms exploits auto-fluorescence following illumination with ultraviolet (UV) light^27^,^28^. Auto-fluorescence is caused by metabolites and structural components of living cells^29^. Although this technique allows continuous real-time monitoring and detection of bioaerosols directly in the air stream, problems remain due to low fluorescence intensity, which leads to poor limits of detection, and requires precise optical systems for the measurements. For these reasons, such devices are typically not portable, and cannot provide an integrated “micro-total analysis system”.

To overcome the limitations discussed above, cell and particle detection methods based on microfluidic flow-cytometry have been reported^30^,^31^,^32^,^33^. Microfluidics benefits from miniaturization, low power consumption and high sensitivity. Microfluidic methods of counting water-borne microorganisms include tracking the population of marine algae and quantification of bacterial cells using pre-staining^34^,^35^,^36^. Chung *et al.* (2014) developed a real-time single-cell detection system using target aptamer-conjugated fluorescent nanoparticles in a microfluidic flow-cytometry platform for microbial diagnostic applications^37^. However, there have been no reports of micro-optofluidic platforms for real-time continuous detection and monitoring of airborne microorganisms.

Here, we demonstrate a micro-optofluidic platform for real-time detection and quantitative analysis of airborne microorganisms. Our optofluidic system involves the following steps: (1) sampling of airborne microorganisms; (2) mixing and reacting in a microchannel for staining; and (3) real-time detection and analysis of the particle by means of light scattering (SC) and bacterial fluorescence (FL). From these optical signals, we may discriminate and quantify airborne microorganisms, enabling simultaneous measurement of the total particle concentration. The performance in terms of particle detection is evaluated using standard particles and three bacterial taxa, and is compared with that of conventional microscopy cell counting and colony counting methods.

## Results

### Design and operation of the micro-optofluidic platform

**Figure 1:**
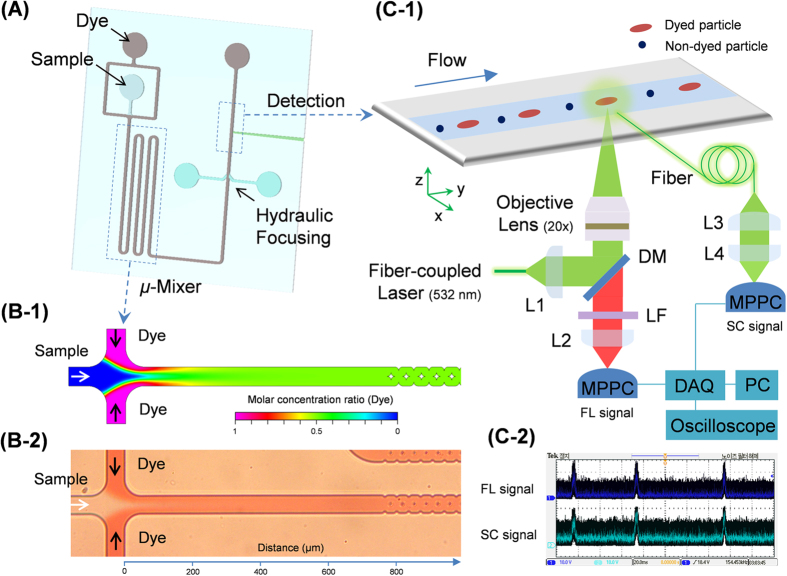
Micro-optofluidic platform for real-time, continuous detection of airborne microorganisms. (**A**) A schematic diagram of the structure of the microchip flow cytometer. It consists of a sample inlet, a micro-mixer, and a detection component in a single layer of PDMS, and fabricated using standard soft lithographic methods. (B-1) Simulated mixing conditions between the sample and the dye in the micro-mixer. The diffusivity coefficient of the dye in water was 10^−9^ m^2^/s. The microfluidic flow rates of both the sample and dye suspension were 12.5 μL/h. The simulations were carried out using CFD-ACE+. (B-2) Optical micrograph of the results corresponding to the simulation shown in B-1. (C-1) Real-time detection of target particles in the core flow in the detection zone, and the optical setup used for continuous particle detection and signal quantification. The target particles in the core of the sample flow were exposed to a fiber-coupled 532-nm laser. The emitted fluorescent (FL) light was collected and brought to the MPPC photo sensor via an optical fiber. A multi-mode fiber was inserted into the PDMS chip to guide the particle scattering (SC) light to the MPPC photo sensor. L1: fiber collimator, f = 7.5 mm; L2: plano-convex lens, f = 50 mm; L3: fiber collimator, f = 18.1 mm; L4: plano-convex lens, f = 25.4 mm; DM: dichroic mirror (552 nm); LF: long-pass filter (560 nm). (C-2) Optical signals for FC and SC monitored using an oscilloscope connected to the MPPC.

Figure 1 shows a schematic diagram of the micro-optofluidic platform. The 150-μm-deep polydimethylsiloxane (PDMS) microfluidic channel was fabricated using conventional soft-lithography processes^38^. The micro-optofluidic platform is based on single-layer PDMS channel and has four inlets (one sample inlet, one dye inlet, and two hydraulic focusing sheath inlets), and one outlet for waste. It consists of four main components: sample and dye inlets, a mixing region, a hydraulic focusing zone, and a sample optofluidic detection component (see Fig. S1). We used SYTO82 dye (Molecular Probes Inc., Eugene, OR, USA) with an excitation wavelength of 541 nm and an emission wavelength of 560 nm for nuclear staining of live cells in the microfluidic mixing zone. SYTO82 provides cell-permeant nucleic acid staining and exhibits low intrinsic fluorescence in cell-free systems, with marked enhancement upon binding to deoxyribonucleic acid (DNA) or ribonucleic acid (RNA)^39^,^40^. Following injection of the sample and dye using a syringe pump (KDS200; KD Scientific Inc., Holliston, MA, USA), the two fluids mixed rapidly at the entrance to the mixing zone.

**Figure 2:**
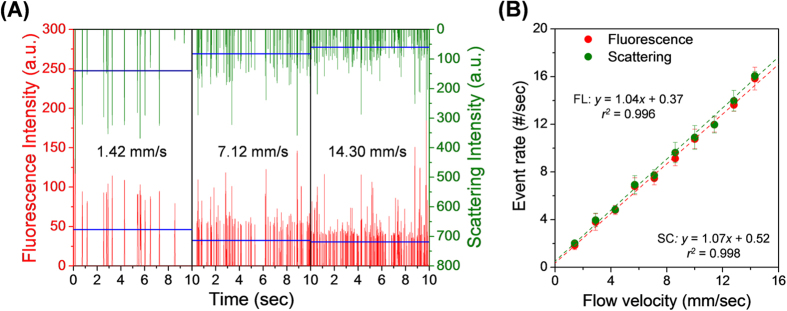
Fluorescence and light-scattering signals of the FLPSL particles. (**A**) Fluorescence and scattering signals acquired using microfluidic flow speeds in the range 1.42–14.3 mm/s. The blue horizontal lines represent average intensities. The microfluidic flow rate of the FLPSL particle suspension in the detection zone was varied in the range 5–50 μL/h, and the sheath flow rate was varied in the range 95–950 μL/h. The ratio of core and sheath flow rates was fixed at 1/20. (**B**) Event rates for fluorescence and scattering signals as a function of particle flow velocity. A linear relationship is evident.

Numerical analysis was carried out using the commercial computational fluid dynamics (CFD) software package CFD-ACE (ESI US R&D Inc., Huntsville, AL, USA) to identify the optimal design and operating conditions. The Navier–Stokes equations were solved with Fick’s law of diffusion to describe the dynamics and homogenization of the mixing process in the channel. Figure 1 (B-1,B-2) show the results of the CFD simulations and optical micrographs of the mixing of the sample and dye in the micro-mixer part. The numerical simulations show that the mixing of the two fluids was complete approximately 0.1–0.4 mm from the entrance of mixing zone with flow rate conditions for each fluid in the range 2.5–25 μL/h (simulation duration, ~72–180 ms) (Fig. S2). The mixing zone was 136.8 mm long and 50 μm wide. The residence time for mixing (and hence for the reaction between the sample and dye) was in the range 25–246 s for total flow rates in the range 5–50 μL/h. After passing through the mixing zone, the mixed sample fluid was focused horizontally using a dual sheath flow with sterilized distilled water (SDW) in the hydraulic focusing component (see Fig. 1 (A)). The focused core flow was 7.5 μm wide, which is one-twentieth the width of the channel. This minimized variation in the optical signals among locations in the particle detection zone^30^.

Figure 1 (C-1) shows a schematic diagram of real-time detection of target particles in the core flow, as well as the optical setup for continuous detection and signal quantification. A diode-pumped solid-state (DPSS) laser with a wavelength of 532 nm and an output power of 2.0 mW (CNI laser, Changchun, China) was coupled with a single-mode optical fiber (core diameter: 3.5 μm, cladding diameter: 125 μm). The laser beam was focused on the core fluid using an objective lens (RMS20x-PF; 20x/0.5; Thorlabs) located beneath the microfluidic channel (~7 μm beam spot size at full-width half-maximum). A highly sensitive multi-pixel photon counter (MPPC) (C10507-11-50U; Hamamatsu Photonics, Hamamatsu City, Japan) module was used to detect the SC and FL signals from the target particles. The particle FL was collected using the objective lens, and entered the MPPC via the optical long-pass filter and focusing lens. To bring the SC light from the particles, multi-mode optical fiber with a core diameter of 105 μm and a cladding diameter of 125 μm (Thorlabs) was carefully inserted into the channel with the axis of the fiber at 90° to the direction of flow, and linked to the MPPC.

A data acquisition (DAQ) unit (NI USB-6356; National Instruments, Austin, TX, USA) was employed to collect the analog data from the MPPC modules, which were recorded using a desktop computer. An oscilloscope (DPO 2012B; Tektronix, Beaverton, USA) was used to monitor the signal in real-time, as shown in Fig. 1 (C-2).

### Performance evaluation of the micro-optofluidic platform using standard PSL particles

The particle detection performance of the optofluidic platform was evaluated under various microfluidic and sample conditions, such that the sample flow rate and the mixing ratios of FL and non-FL particles were varied. We used standard spherical polystyrene latex (PSL) and fluorescence PSL (FLPSL) particles that were 1 μm in diameter.

Figure 2 shows the particle FL and SC signals with flow velocities in the range 1.42–14.3 mm/s. The FLPSL particle suspension (~1.45 × 10^3^/μL) was injected into sample inlet. The ratio of the flow rate of the FLPSL particle suspension to that of the SDW was 1:19 in the narrow core region (which had dimensions of 7.5 × 150 μm). As the flow velocity increased from 1.42 to 14.3 mm/s, the frequency of the particle SC and FL signals increased in proportion to the particle flow velocity, and the average intensity of the measured SC and FL signals (SC/FL) decreased from ~140 arbitrary unit (a.u.)/~60 a.u. to ~46 a.u./~30 a.u., respectively (Fig. 2 (A)). This is attributed to the decreased photon integration time of the photodetector with decreasing residence time of the particles in the detection zone of microchannel^41^. Figure 2 (B) shows that the event rate (i.e., number of counts per second) of the particle SC and FL signals followed a linear relationship with the particle flow velocity; for FL we have *y* = 1.04*x* + 0.37, with *r*^2^ = 0.996, and for SC we have *y* = 1.07*x* + 0.52, with *r*^2^ = 0.998. The index of coincidence between the total frequencies of particle SC and FL signals was ~96.5% over the entire range of particle flow velocities. McClain *et al.* reported that only ~5% of the particle SC signals were unaccompanied by coincident FL signals, which was attributed to unresolved FL peaks due to the relatively low signal intensity^36^. When the number of counts of the particle SC and FL signals are plotted as histograms as a function of the signal intensity, both sets of signals form Gaussian-like distributions, as shown in Fig. S3. The variation in the intensity results from variations in the particle size and particle position in the detection zone. As expected, the particle SC signal was significantly stronger than the FL signal.

**Figure 3:**
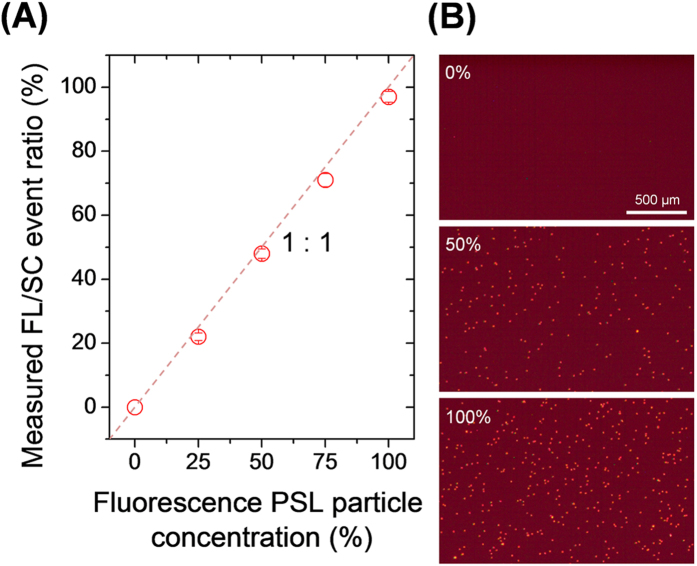
Fluorescence to scattering signal ratio (FL/SC) for various PSL and FLPSL particle mixture conditions. (**A**) Measured fluorescence to scattering signal ratio (FL/SC). The result was proportional to the mixing ratio of 1-μm-diameter PSL and FLPSL particles; the dotted line indicates a ratio of 1:1. The flow rate of both the PSL and FLPSL particle suspensions was 12.5 μL/h at the sample and dye inlets, and was 237.5 μL/h in the sheath flow. The total microfluidic flow rate in the particle detection region was 7.1 mm/s. (**B**) Fluorescence micrographs of the FLPSL particles with various mixing ratios. The FLPSL particle number increased linearly with the FLPSL concentration.

We evaluated the particle detection performance of the micro-optofluidic platform with various mixing ratios of FLPSL and PSL particles. The total particle concentration was fixed at ~1.45 × 10^3^/μL, and the total flow rate of the mixed suspension was 25 μL/h, giving a flow velocity of ~7.1 mm/s. The mixing ratio of FLPSL and PSL particle suspensions was varied in the range 0 – 100%. As shown in Fig. 3 (A), the ratio of the count rate of FL signals to SC signals increased in proportion to the mixing ratio of FLPSL particles. Figure 3 (B) shows fluorescence microscopy images of the FLPSL particles in the mixing suspension. When the mixing ratio of FLPSL particles was 0%, the FLPSL particle number concentration in the micrographs was 0/μL; at a mixing ratio of 50%, the FLPSL particle number concentration was 688/μL (~95% of the initial FLPSL particle concentration); and at a mixing ratio of 100%, the FLPSL particle number concentration was 1406/μL (~97% of the initial FLPSL particle concentration). PSL particles could not be enumerated using normal bright-field microscopy because of the limit of resolution and small field of view.

From this performance evaluation (Figs 2 and 3), we may conclude that the micro-optofluidic platform demonstrated not only real-time and continuous particle detection, with suitable performance for standard PSL particles, but also quantitative and accurate discrimination between FL and non-FL particles.

### Real-time detection of airborne microorganisms

To simulate a hazardous airborne bacteria-contaminated environment, we prepared a 1 × 1 × 1 m test chamber containing live, airborne *Escherichia coli*, *Bacillus subtilis* and *Staphylococcus epidermidis* bioaerosols separately. Details of the experimental setup can be found in the Methods and Supplemental Information (Fig. S4).

**Figure 4:**
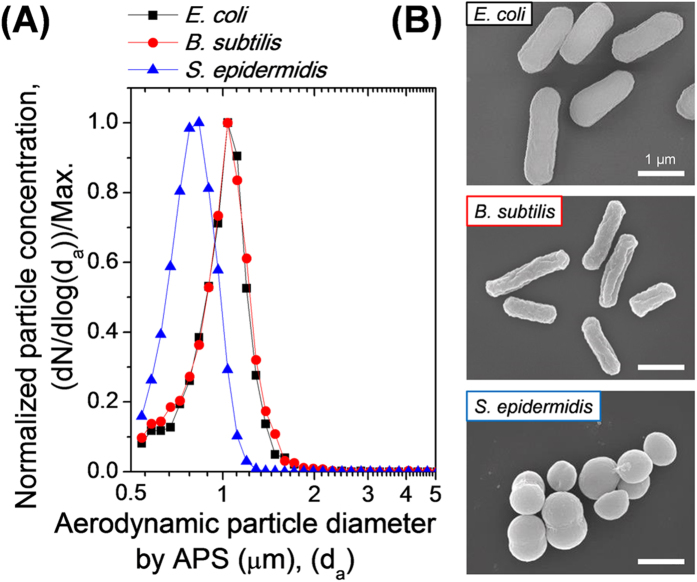
The size and morphology of the test airborne bacterial particles. (**A**) The normalized aerodynamic particle size distributions of *E. coli*, *B. subtilis*, and *S. epidermidis* bioaerosols. The aerodynamic diameter is equivalent to the diameter of standard-density spherical particles with the same gravitational settling velocity as the original particles^48^. The number concentration of the particles in air was recorded for each APS channel size, divided by the logarithmic interval of the corresponding particle size range and plotted as a function of the aerodynamic diameter^48^. The particle number concentrations were then normalized to the highest concentration for each bacterium. (**B**) SEM images of *E. coli* and *B. subtilis* cells (rod-shaped) and *S. epidermidis*, cells of which are spherical and smaller than those of the other two bacteria.

Figure 4 shows the size and morphology of the bacterial bioaerosols in the test chamber. Real-time particle size distribution data of the bacterial bioaerosols were obtained using an aerodynamic particle sizer (APS; 3321; TSI Inc., Shoreview, MN, USA). As shown in Fig. 4 (A), the bacterial bioaerosols exhibited mono-modal curves with a specific geometric mean diameter (GMD), peak diameter, and geometric standard deviation (GSD). Table 1 lists these data for the three bioaerosols; the GMDs of *E. coli* and *B. subtilis* were similar at ~1 μm, and that of *S. epidermidis* was somewhat smaller, at ~0.8 μm.

Airborne bacterial bioaerosols in the test chamber were collected using a BioSampler (SKC Inc., Eighty Four, PA, USA), which is a highly efficient collection device that traps airborne microorganisms in a swirling liquid for subsequent analysis^42^. The airborne particle collection efficiency of the BioSampler was ~94.3% for the 1-μm-diameter PSL particles (Fig. S5). Figure 4 (B) shows scanning electron microscope (SEM) images of the bioaerosols collected using the BioSampler. *E. coli* and *B. subtilis* exhibited rod-shaped morphology, with diameters in the range 0.6 – 0.8 μm and lengths in the range 0.8 – 1.5 μm. *S. epidermidis* cells were spherical, and 0.6 – 1.0 μm in diameter.

**Figure 5:**
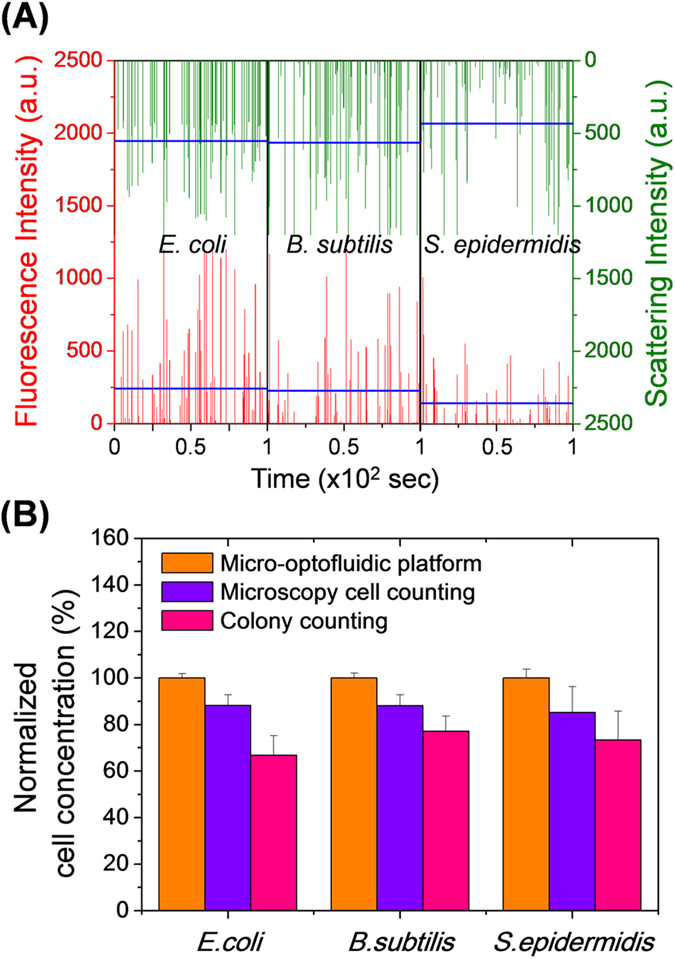
Detection and quantification of airborne microorganisms using the micro-optofluidic platform. (**A**) Fluorescence and scattering signals acquired for *E. coli*, *B. subtilis*, and *S. epidermidis*. The microfluidic flow rate was 12.5 μL/h at both the sample and dye inlets, and was 237.5 μL/h for the sheath flow. The data were recorded for a total of 100 s. The blue horizontal lines indicate the mean intensities. (**B**) Bacterial cell concentrations measured using the micro-optofluidic platform, cell counting using fluorescence microscopy, and colony counting. The cell concentrations measured by the optofluidic platform were normalized to the fluorescence signal event rate.

Samples containing the bacterial suspension were injected into the micro-optofluidic platform using a syringe pump to evaluate the device performance for the real-time detection of airborne bacterial particles. Figure 5 (A) shows the acquired SC and FL signals of the test bacterial bioaerosols. The average SC/FL signal intensity ratios were ~555/240 for *E. coli*, ~565/227 for *B. subtilis*, and ~433/142 for *S. epidermidis*. Because of the size of these bacteria (i.e., 0.5 – 1.6 μm), they may be considered to lie within the Mie scattering regime, and the intensity of the SC signals may be assumed to be proportional to the cross-sectional area of particles^36^. In the same manner, the FL signal intensity increased proportionally with the size of the bacteria because larger particles have a higher concentration of nucleic-acid fluorescent dye^33^,^36^. The particle number concentration was determined based on the frequency data for the SC and FL signals. Table 2 lists the SC signal concentration, which corresponds to the total particle number concentration. This was higher than the FL signal concentration, which corresponds to the total concentration of microorganisms. The difference between the total particle count (SC signals) and the total bacterial concentration (FL signals) was ~7% for *E. coli*, ~16% for *B. subtilis*, and ~23% for *S. epidermidis*. It follows that the sample included other particles; i.e., non-microorganism particles or impurities, such as non-dyed particles or small debris.

To evaluate the particle detection efficiency for airborne microorganisms, the total concentration of bacteria obtained from the FL signal of the micro-optofluidic platform was compared with the conventional fluorescence microscopy cell counting and colony counting methods. Figure 5 (B) shows a comparison of the concentration of bacteria measured using fluorescence microscopy and colony counting, both of which were normalized to that measured using the micro-optofluidic platform. For *E. coli* the normalized cell concentration by microscopic cell counting was 88 ± 4.6%, and was 67 ± 8.5% using colony counting; for *B. subtilis*, the normalized cell concentration using microscopic cell counting was 88 ± 4.7%, and was 77 ± 6.6% using colony counting; and for *S. epidermidis* the normalized cell concentration using microscopic cell counting was 85 ± 11.1%, and was 73 ± 12.4% using colony counting. The concentration of bacteria measured using both conventional cell-counting methods was lower than that using the micro-optofluidic platform.

The micro-optofluidic platform exhibited the highest cell counts and the lowest standard deviation, which is indicative of superior performance. Photobleaching of fluorophores may occur during cell counting via conventional fluorescence microscopy, which results in a reduction in the fluorescence intensity. Furthermore, the low signal-to-noise ratio in the imaging process may lead to underestimation of the number of cells. An inappropriate image threshold intensity setting is required to discriminate fluorescence particles from the background, and this may exclude particles that either have a low fluorescence intensity or are slightly out of focus^43^. Furthermore, quantitative analysis of the total concentration of particles with sizes of less than 1 μm is limited by the resolution and the field of view of bright-field microscopy. As shown in Figure 5 (B), the colony counting method resulted in the lowest cell concentration of the three methods. The colony counting method measures only culturable cells in the medium. Therefore, it is difficult to apply the colony counting method for the analysis of viable but non-culturable (VBNC) microorganisms, or microorganisms that require a specific growth environment and/or specific nutrients^44^.

The micro-optofluidic platform with integrated sample preparation and detection simplifies the system, can significantly reduce the measurement time, and can yield more accurate quantitative results compared with the abovementioned conventional methods. Furthermore, our system provides additional information on the total particle number concentration of aerosols, which is difficult to obtain using conventional methods.

## Discussion

We have demonstrated continuous, rapid and real-time detection of bioaerosols using a micro-optofluidic platform. The performance of our device was investigated using standard PSL particles with various flow speeds and mixing ratios with FLPSL particles. Accurate quantitative FLPSL particle discrimination with high efficiency was achieved. This first application of this integrated micro-optofluidic platform for the analysis of airborne microorganisms showed that our device could reduce the time for sample preparation and manual analysis compared with conventional microorganism-counting methods. The micro-optofluidic platform has potential applications in aerosol analysis, and can enable portable, highly sensitive, continuous real-time detection of airborne particles and microorganisms.

In future, we plan to include a 3D focusing stream to optimize the optical signals, which is expected to enhance the resolution and efficiency of the measurements, and to reduce the noise due to spatial deviations of the particles. Accurate particle sizing using the current micro-optofluidic platform is possible^30^; furthermore, a micro-pump between the BioSampler and microfluidic channel could be included for continuous real-time analysis of airborne microorganisms. Various species of dye or marker for specific target materials, such as target-specific antibodies or aptamers, could be used to create an analysis system that provides data on the physical, chemical and biological properties of the airborne particles^37^. Note that the purpose of this work was to quantitate airborne microorganisms rapidly and in real-time using a fully integrated micro-optofluidic platform.

## Methods

### Sample preparation

Two types of standard uniform particles and three types of bacteria were used in this study. Fluorescence polystyrene latex (FLPSL) particles (F8820; Fluorescent microsphere; 1.0-μm diameter; orange fluorescent (540/560); Invitrogen) and polystyrene latex (PSL) particles (4010A; Monosized microsphere; 1.0-μm diameter; refractive index of 1.59; density of 1.06 g/cm^3^; Duke Scientific Corporation) were used as standard particles for the performance evaluation of particle detection and quantification. As test airborne microorganisms, Gram-negative *Escherichia coli* (Korean Collection of Type Cultures (KCTC) 1039, Biological Resource Center, Republic of Korea), Gram-positive *Bacillus subtilis* (KCTC 1022), and Gram-positive *Staphylococcus epidermidis* (ATCC 12228), were used^45^. The bacteria were incubated in nutrient broth (Becton Dickinson, Franklin Lakes, USA) at 37 °C, and harvested using a centrifuge (Mini, Gyrozen, South Korea) at 6000 rpm for 10 minutes. Bacterial pellets were washed three times using SDW with a centrifuge to remove the residual medium. To create the bacteria suspensions, 30-mL aliquots were placed in a six-jet collision nebulizer (BGI Corp., USA) to aerosolize the bacteria.

### Microchannel design and fabrication

The microchannel was a single-layer PDMS channel fabricated using conventional soft lithography. SU-8 (2025; Microchem Corp.), which is a negative photoresist, was spin-coated onto a 4.2-inch Si wafer. The photoresist was soft baked on a hotplate at 65 °C for 5 min, followed by 95 °C for 10 min. A chrome photomask pattern was installed using a UV aligner, and the photoresist was exposed to UV irradiation. Following this exposure, the resist was baked at 65 °C for 2 min and then at 95 °C for 10 min. The SU-8 photoresist pattern was developed using 1-methoxy-2-propyl acetate (Microchem Corp.) for 7 min. After completion of the developing process, the pattern was rinsed using isopropyl alcohol (IPA) and deionized (DI) water to yield a master mold. PDMS was poured over the developed wafer with the pattern, and cured in an oven at 65°C. The patterned PDMS channel was then removed from the wafer, and bonded onto glass using O_2_ plasma to seal the microchannel.

### Collection of airborne microorganisms

A BioSampler® (SKC Inc., Eighty Four, PA, USA) was used to collect bacterial bioaerosol samples in the test chamber. The test bioaerosols were collected in 20 mL of phosphate-buffered saline (PBS) at pH 7.0. Bacterial bioaerosols with a nominal flow rate of 12.5 L/min were collected with a sampling time of 15 min.

### Fluorescence microscopy cell counting

To enumerate FLPSL particles and bacterial cells using the fluorescence microscopy method, 10–20 μL of sample (following enrichment via centrifugation in the case of low particle concentrations) was loaded into the sample injection area of a disposable hemocytometer (DHC-N01; INCYTO, Republic of Korea), and visualized using a fluorescence microscope (B X 51; Olympus, Tokyo, Japan) with a U-MWG2 filter set; the excitation wavelength was in the range 510–550 nm, and the emission wavelength was >590 nm. For each sample, images of at least 16 microscopic fields were captured using a CCD array camera. Microscopy cell counting was carried out using the ImageJ software package (http://imagej.nih.gov/ij/).

### Colony counting

Bacterial suspensions were serially diluted and spread onto the surface of nutrient agar (Becton Dickinson) in a petri dish, followed by incubation at 37°C for 24 hours. The resulting colonies were counted manually.

### Scanning electron microscopy

The morphology of airborne bacterial particles was investigated using SEM (Nova Nano SEM 200; FEI Co., Hillsboro, OR, USA). The modified Karnovsky’s fixation protocol was used^46^,^47^, and bacterial samples were fixed using 2% paraformaldehyde (15713; Electron Microscopy Sciences (EMS), Hatfield, PA, USA) and 2% glutaraldehyde (16020; EMS) in a 0.05 M sodium cacodylate buffer (SCB; pH 7.2) (11652; EMS) at 4°C for 2 hours, followed by washing with 0.05 M SCB at 4°C for 2 hours. Post-fixation, the samples were treated with 1% osmium tetroxide (19152; EMS) in 0.05 M SCB at 4°C for 2 hours, and then washed with distilled water at room temperature. The samples were dehydrated at room temperature in a 30–100% ethanol series with 10-minute exposure to each concentration. The ethanol was then replaced with hexamethyldisilazane (16700; EMS). Following dehydration, each sample was spread onto polycarbonate membrane filters with a pore size of 0.4 μm (Isopore Membrane Filters HTTP01300; Millipore, Billerica, MA, USA) and coated with osmium by chemical vapor deposition (HPC-1SW; Vacuum Device Inc., Ibaraki, Japan).

## Additional Information

**How to cite this article**: Choi, J. *et al.* Integrated micro-optofluidic platform for real-time detection of airborne microorganisms. *Sci. Rep.* **5**, 15983; doi: 10.1038/srep15983 (2015).

## Supplementary Material

Supplementary Information

## Figures and Tables

**Table 1:** Size characteristics of test airborne bacterial particles.

Bacterium	Airborne microorganisms		
	GMD (μm)	Peak diameter (μm)	GSD
*E. coli*	0.98 ± 0.004	1.04 ± 0.001	1.24 ± 0.341
*B. subtilis*	0.99 ± 0.013	1.04 ± 0.030	1.26 ± 0.013
*S. epidermidis*	0.79 ± 0.002	0.84 ± 0.026	1.18 ± 0.001

The geometric standard deviation (GSD) is defined as 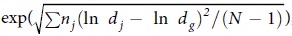, where *d*_*j*_ is the diameter of an individual particle, *n*_*j*_ is the number of particles in the *j*^*th*^ group, *N* is the total number of particles, and ln *d*_*g*_ is the natural logarithm of the geometric mean diameter (GMD) of the particles, defined as 
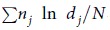
. Data are listed as the means of 10 repetitions plus/minus the standard deviation.

**Table 2:** Numbers of test bacteria per unit volume (/μL) determined using the micro-optofluidic platform, and by microscopy cell counting and colony counting. Data are the means of 5 repetitions plus/minus the standard deviation.

Bacterium	Micro-optofluidic platform		Microscopy cell counting	Colony counting
	Total particle (SC^a^)	Total bacteria (FL^b^)		
*E. coli*	10.6 ± 0.73	9.9 ± 0.18	8.7 ± 0.45	6.6 ± 0.84
*B. subtilis*	9.3 ± 0.63	7.8 ± 0.17	6.9 ± 0.37	6.0 ± 0.52
*S. epidermidis*	8.0 ± 0.63	6.5 ± 0.25	5.6 ± 0.73	4.8 ± 0.81

^a^Particle scattering

^b^Fluorescence.
