# Supplementary material for: Integrated micro-optofluidic platform for real-time detection of airborne microorganisms
Source: Sci Rep. 2015 Nov 2;5:15983. doi: 10.1038/srep15983 (PMC4629162; doi:10.1038/srep15983)
Supplement: Supplementary Information [file srep15983-s1.doc]

**Supplementary Information for**

**“Integrated micro-optofluidic platform for real-time detection of airborne microorganisms”**

Jeongan Choi**^†^**, Miran Kang**^†^**, and Jae Hee Jung*

Center for Environment, Health, and Welfare Research, Korea Institute of Science and Technology, Seoul 136-791, Republic of Korea

**^†^**Authors equally contributed to this work.

*Correspondence should be addressed to: [jaehee@kist.re.kr](mailto:jaehee@kist.re.kr); Tel.: 82-2-958-5718

**Figure S1**


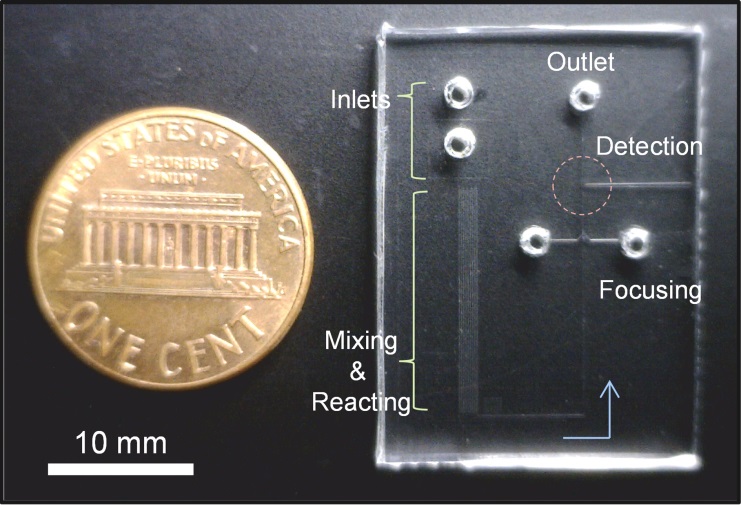


**Fig. S1. Optical photograph of the micro-optofluidic chip.** The microfluidic chip consisted of a sample inlet, micro-mixer, and detection components, and was formed in a single layer of PDMS fabricated using standard soft photolithographic methods. The dimensions of the chip were 29 × 22 × 5 mm; a US one-cent (penny) coin is shown to indicate scale.

**Figure S2**

**Fig. S2. Numerical simulation of mixing of the sample and dye fluids under sample and dye flow rates of (A) 2.5 μL/h and (B) 25 μL/h.** The effects of diffusion on the mixing can be represented by the Peclet number, $Pe=UL/D$, where $U$ is the inlet velocity, $L$ is the hydraulic diameter of the channel, and $D$ is the diffusivity. The diffusivity of dye in water was 10^-9^ m^2^/s. $Pe$ describes the ratio of the longitudinal convection time to the lateral diffusion time. A Smaller Peclet number indicates more rapid and uniform mixing.^1,2^ The numerical simulations showed that the mixing of two fluids was complete ~0.1 mm from the mixing zone with $Pe$ = 27.8, and (see Fig. S2 (A)) and ~0.4 mm from the mixing zone with$Pe$ = 278 (see Fig. S2 (B)). The time for mixing was ~72 ms with a flow rate of 2.5 μL/h for both the sample and dye (see Fig. S2 (B)) and ~180 ms with flow rate of 25 μL/h for both sample and dye (Fig. S2 (A)). The length of the mixing zone was ~136.8 mm and the width was 50 μm. The residence time for mixing and reaction between the sample and dye was 25–246 ms with a total flow rate of 5–50 µL/h.

**Figure S3**

**Fig. S3. (A) Size distribution of FLPSL particles, histograms of (B) the scattering, and (C) fluorescence intensities of FLPSL particles with a flow rate of 14.3 mm/s.** FLPSL particles have a peak value of 1 µm. Both SC and FL signals exhibited clear peak intensities. The particle SC signal was stronger than the FL signal. Both signals exhibited narrow intensity variations, with Gaussian-like distributions similar to the size distribution of the FLPSL particle. The coefficients of variation (CV) were ~24.4% for the SC signal and ~13.6% for the FL signal. The variation in the intensity is attributed mainly to variation in the particle size distribution and position within the detection zone.

**Figure S4**

**Fig. S4. Schematic diagram of the experimental setup used for the real-time detection of airborne microorganisms.** The test bacterial pellets were washed three times in sterilized deionized water (SDW) and centrifuged at 5000 *g* for 10 min. The pellets were then diluted to an optical density of ~0.89 at 600 nm. Aliquots (30 mL) were removed and placed in a Collison nebulizer (BGI Inc., Waltham, MA, USA). The cell concentration was approximately 10^8^ colony-forming units (CFU) per mL. Bacterial particles were aerosolized from a liquid suspension using a nebulizer stem at a flow rate of 5 L/min with dry, filtered, compressed air, and the aerosolized bacterial particles were passed through a diffusion dryer and a ^210^Po neutralizer for moisture removal and to neutralize electronic charge. The bacterial bioaerosols were diluted using additional air with a flow rate of 30 L/min, and introduced into the 1 × 1 × 1-m test chamber using a fan-type air mixer. The particle size distribution and bioaerosol concentrations were measured using an aerodynamic particle sizer (APS; 3321, TSI Inc.), which sizes airborne particles in the range 0.5–10 μm using a time-of-flight (TOF) technique and measures the aerodynamic diameter in real time. The aerodynamic diameter of a particle is equivalent to that of standard-density spherical particles with the same gravitational settling velocity.^3^ A BioSampler (SKC Inc., Eighty Four, PA, USA) was used for bacterial bioaerosol sampling in the test chamber. The test bioaerosols were collected in 20 mL of phosphate-buffered saline (PBS) at pH 7.0. Aliquots of BioSampler suspension were used for colony counting and fluorescence microscopy cell counting, as well as scanning electron microscopy analysis. This suspension was also introduced into the microchannel of the optofluidic flow cytometry system using a syringe pump.

**Figure S5**

**Fig. S5. Collection efficiency of standard PSL particles in the BioSampler.** The BioSampler (SKC Inc.) is a highly efficient bioaerosol collection device that traps airborne microorganisms in a swirling liquid for subsequent analysis. The BioSampler was formed of glass and consisted of three parts: an inlet, a nozzle section with three tangential sonic nozzles, and a collection vessel. The collection vessel was filled with a liquid collection medium. The nozzles of the BioSampler create a swirling airflow that maintains microorganism viability by gently moving particles onto the collection surface without re-aerosolization.^4^ Figure S5 shows the collection efficiency as a function of the aerodynamic particle diameter for the BioSampler, with flow rate of 12.5 L/min. The aerodynamic particle diameter is defined as $d_{e}\left(\rho_{p}/(\rho_{0}\cdot\chi)\right)^{1/2}$, where $d_{e}$ is the equivalent volume diameter of particle, $\rho_{p}$ and $\rho_{0}$ are real particle density and standard particle density (1 g/cm^3^), respectively, and $\chi$ is the dynamic shape factor; i.e., the diameter of a spherical particle with a density of 1 g/cm^3^ that has the same settling velocity, and standardizes for shape and density to enable wide application in aerosol technology.^3^ The collection efficiency of the 1-μm PSL particles was ~94.3%.

**Reference**

1 Maeng, J., Yoo, K., Song, S. & Heu, S. Modeling for fluid mixing in passive micromixers using the vortex index. *J. Korean Phys. Soc.* **48**, 902-907 (2006).

2 Nguyen, N.-T. & Wu, Z. Micromixers—a review. *J. Micromech. Microeng.* **15**, R1-R16 (2005).

3 Hinds, W. C. *Aerosol Technology: Properties, Behavior, and Measurement of Airborne Particles*. (Wiley-Interscience, 1999).

4 Willeke, K., Lin, X. & Grinshpun, S. A. Improved aerosol collection by combined impaction and centrifugal motion. *Aerosol Sci. Technol.* **28**, 439-456 (1998).
